# Use of whole-exome sequencing to identify novel monogenic gene mutations and genotype–phenotype correlations in Chinese Han children with urolithiasis

**DOI:** 10.3389/fgene.2023.1128884

**Published:** 2023-04-18

**Authors:** Zhi Wang, Tianqu He, Li liu, Fangyun Tong, Chuangye Li, Yaowang Zhao, Yanfang Li

**Affiliations:** Department of Urology, Hunan Children’s Hospital, Changsha, Hunan, China

**Keywords:** pediatric urolithiasis, Chinese population, monogenic disease, whole-exome sequencing, metabolic evaluation

## Abstract

The incidence of urolithiasis (UL) in children has been increasing. Although the pathogenesis of pediatric UL is controversial and remains unclear, multiple monogenic causes of UL have been identified. We aim to investigate the prevalence of inherited UL causes and explore the genotype–phenotype correlation in a Chinese pediatric group. In this study, we analyzed the DNA of 82 pediatric UL patients using exome sequencing (ES). The data of metabolic evaluation and genomic sequencing were subsequently analyzed together. We detected 54 genetic mutations in 12 of 30 UL-related genes. A total of 15 detected variants were described as pathogenic mutations, and 12 mutations were considered likely pathogenic. Molecular diagnoses were made in 21 patients with pathogenic or likely pathogenic variants. Six novel mutations that were not previously reported were identified in this cohort. Calcium oxalate stones were detected in 88.9% cases (8/9) with hyperoxaluria-related mutations, while 80% of individuals (4/5) with cystinuria-causing defects were diagnosed with cystine stones. Our study highlights the significant genetic abnormalities in pediatric UL and demonstrates the diagnostic power of ES for screening patients with UL.

## 1 Introduction

Urolithiasis (UL) is a highly prevalent health problem worldwide, affecting 4%–20% of all individuals (Quhal and Seitz, 2021). It is associated with significant morbidity because of frequent recurrence, the necessity for surgical intervention, and progression to chronic kidney disease, which heavily burdens the development of social healthcare (Rule et al., 2009). Patients with UL usually present with renal colic, hematuria, urinary obstruction, and urinary tract infections that can aggravate renal dysfunction and cause kidney loss. The specific risk of developing UL varies according to geography, dietary structure, socioeconomic status, and genetic factors (Monico and Milliner, 2011).

Significant increases in the incidence and prevalence of UL in adult populations have been observed for decades (Turney et al., 2012). Although UL is less prevalent in children and adolescents, the annual incidence of pediatric nephrolithiasis has increased by 4%–10% over the last two decades (Dwyer et al., 2012; Alfandary et al., 2018). The unique congenital deformities, anatomical structures, and metabolic patterns contribute to a higher UL recurrence rate in children than in adults. Among patients diagnosed with UL during childhood, there is a 50% probability of recurrence within 3 years (Tasian et al., 2017). Various factors are involved in the development of nephrolithiasis, including fluctuations in urinary pH, inadequate liquid intake, medications, metabolic abnormalities such as hypercalciuria and hyperoxaluria, and hereditary disorders (Miah and Kamat, 2017). However, the cause of UL has not yet been elucidated.

In pediatric patients, genetic and anatomical causes account for up to 75% of the risk factors for UL development (van’t Hoff, 2004). A positive family history of UL has been reported in 30%–80% of cases (Scoffone and Cracco, 2018). A cohort study estimated that the relative risk for UL was 2.57-fold in individuals with a positive family history compared to those with a negative family history (Curhan et al., 1997). Genetic or metabolic alterations usually lead to remarkable changes in serum or urine components, disrupting the balance between pro- and anti-lithogenic substances in body fluids (Hoppe and Martin-Higueras, 2020)^.^ According to the Online Mendelian Inheritance in Man (OMIM) catalog, monogenic mutations in 30 known nephrolithiasis (NL)-causing genes can contribute to the formation of NL by autosomal recessive, autosomal dominant, or X-linked transmission. These mutations were detected in 20.8% of the children with the onset of NL (Halbritter et al., 2015). Metabolic disorders involved in nephrolithiasis have been identified in more than 75% of cases. Hypercalciuria and hypocitraturia are the most common metabolic disorders in UL (Bowen and Tasian, 2018). Because pediatric patients with UL are more susceptible to monogenic alterations, genetic sequencing is particularly critical in patients with early disease onset because of its benefits in tailoring personal treatment regimens and follow-up. Misdiagnosis and missed diagnosis of inherited UL usually occur because of the lack of an effective genetic mutation detection approach.

Recently, exome sequencing (ES), as a breakthrough next-generation technique, has offered an in-depth and comprehensive way to detect potential pathogenic mutations at a relatively low cost and in a short time and is regarded as a cost-effective screening method (Aggarwal, 2021). Although a growing number of studies have outlined the prevalence of monogenic mutations and novel disease-causing loci (Halbritter et al., 2015; Daga et al., 2018; Wang et al., 2020), the contribution of monogenic causes of UL has not been extensively studied in Chinese pediatric patients. In the present study, we used ES to screen 82 children with UL for hereditary causes. Six novel mutations were identified in 6 patients. In addition, metabolic evolution and stone component analyses were performed to clarify the genotype–phenotype correlations in the pediatric UL group.

## 2 Materials and methods

### 2.1 Study cohort and data collection

This study was approved by the Institutional Review Board of Hunan Children’s Hospital (No. HCHLL-2021-14). We conducted a retrospective study of data from subjects diagnosed with pediatric urolithiasis based on a combination of clinical manifestations and imaging diagnoses (ultrasound, CT, or radiography). Patients with conditions or medications that may lead to secondary UL were excluded. A total of 82 pediatric patients enrolled between January 2021 and March 2022 consented to undergo ES after being informed of the study aim, the importance of ES, and data publication. Informed consent was obtained from all guardians of the subjects. Demographic characteristics, 24-h urinalysis (24-h urine oxalate, citrate, and calcium), imaging evidence, and stone composition data were collected for further analysis. The stone composition was analyzed using an automatic infrared spectrum analysis system, LIIR-20 (approved by the Chinese FDA, No. 2008-2210004). Metabolic diagnosis was made through 24-h urine sample results in accordance with the previously published criteria (Bevill et al., 2017; Bowen and Tasian, 2018) and the EAU Guideline on Pediatric Urology 2022 (Radmay et al., 2022). Monogenetic urolithiasis was diagnosed based on a combination of clinical evidence (imaging findings, metabolic evaluation, and calculus composition analysis) and genetic mutation sequencing. Metabolic analysis results were not available for all subjects owing to a lack of urine or stone samples (diaper use or no immediate expulsion after extracorporeal shockwave lithotripsy).

### 2.2 Mutation screening and analysis

Genomic DNA was extracted from the peripheral blood lymphocytes of the patient and corresponding parents using a genomic DNA isolation kit (D3392-02; Omega Bio-Tek, Inc., Norcross, GA, United States) according to a standard protocol. All DNA samples were captured using the Agilent SureSelect Human All Exon V5 Kit (Agilent, California, United States), followed by exome sequencing on an Illumina NextSeq 550 platform (Illumina Inc., San Diego, United States). FASTQ data from the sequencing platform were obtained using Bcl2fastq (v2.0.1), and the acquired data were processed using Trimmomatic (version 0.36) to remove low-quality reads, bases, and trimming adaptors. The sequencing reads were aligned to the NCBI human reference genome (gh19/NCBI37.1). Variant calling was performed using the Genome Analysis Toolkit. Finally, the variant call format files were analyzed using ANNOVAR tools. A total of 30 known monogenic causes of UL genes (defined by OMIM; www.ncbi.nlm.nih.gov/omim) (Braun et al., 2016) were particularly screened to identify underlying variants in UL pediatric patients.

### 2.3 Evaluation and prediction of pathogenicity of detected mutation

Prior to the analysis, variants were screened using the Human Gene Mutation Database (HGMD; http://www.hgmd.cf.ac.uk/ac/index.php), dbSNP database (dbSNP; http://www.ncbi.nlm.nih.gov/SNP), gnomAD browser (http://gnomad.broadinstitute.org/), and ClinVar database (ClinVar; http://www.ncbi.nlm.nih.gov/clinvar) as public references. Pathogenicity of genetic mutations was evaluated according to the American College of Medical Genetics Genomics (ACMG) and classified as “pathogenic,” “likely pathogenic,” and “uncertain” (Amendola et al., 2016). Molecular diagnosis was made in cases carrying variants that were classified as “pathogenic” or “likely pathogenic” according to the ACMG standard, which we defined as “positive cases.” Cases with “uncertain variants” were defined as “uncertain cases,” and patients without any UL-related genetic mutation detected were considered “negative cases.” The deleterious effects of missense variants were predicted using the PolyPhen-2 (Adzhubei et al., 2010) (http://genetics.bwh.harvard.edu/pph2/), PROVEAN/SIFT (Choi et al., 2012) (http://provean.jcvi.org/index.php), MutationTaster (Schwarz et al., 2014) (https://www.mutationtaster.org), and Franklin by Genoox (https://franklin.genoox.com) algorithms.

## 3 Results

### 3.1 Study population characteristics

From January 2021 to March 2022, we enrolled a cohort of pediatric stone patients across sex, origin, residence, and nephrolithiasis history, as evidenced by the demographic data of the probands from these families. None of the patients had secondary reasons for UL, including the use of specific medications, congenital anatomical abnormalities, or gastrointestinal absorption dysfunction. The clinical features of the 82 patients who underwent exome sequencing are summarized in Table 1. In total, 55 patients were male, while 27 were female (M:F = 2.03:1). The median age of onset was 3 years (Range 0.17–15 years). All patients were of Han Chinese ethnicity. Stone locations were also recorded. The distribution of stones detected in the kidneys, ureters, and bladders was 82.9% (68/82), 15.9% (13/82), and 1.2% (1/82), respectively. A total of 49 patients who completed daily urine collection underwent 24 h urine oxalate, calcium, and citrate evaluation. Patients with multiple metabolic abnormalities were included in each subgroup. Based on the available 24 h urine excretion component analysis, 15 (30.6%) probands met the criteria for hyperoxaluria, 35 (71.4%) had detectable decreased uric citrate levels, and 19 (38.8%) had elevated urine calcium excretion above the normal threshold. Infrared spectrum analysis for stone composition identification was applied to 59 patients; calcium oxalate stone was the most predominant calculus, accounting for 54.2% (32/59), followed by cystine in nine patients (15.3%), carbonate apatite in eight (13.6%), uric acid in seven (11.9%), purine in two (3.4%), and calcium phosphate in one patient (1.7%).

**TABLE 1:** Clinical features of the cohort.

Characteristic	Description
Total no. in study	82
Onset age (year), median (range)	3 (0.17–15)
Gender, *n* (%)	
Male	55 (67.1)
Female	27 (32.9)
Stone location, *n* (%)	82
Kidney	68 (82.9)
Ureter	13 (15.9)
Bladder	1 (1.2)
Metabolic evaluation, *n* (%)	49
Hyperoxaluria	15 (30.6)
Hypocitraturia	35 (71.4)
Hypercalciuria	19 (38.8)
Stone composition, *n* (%)	59
Calcium oxalate	32 (54.2)
Cystine	9 (15.3)
Carbonate apatite	8 (13.6)
Uric acid	7 (11.9)
Purine	2 (3.4)
Calcium phosphate	1 (1.7)

### 3.2 Genetic mutation findings

**FIGURE 1:**
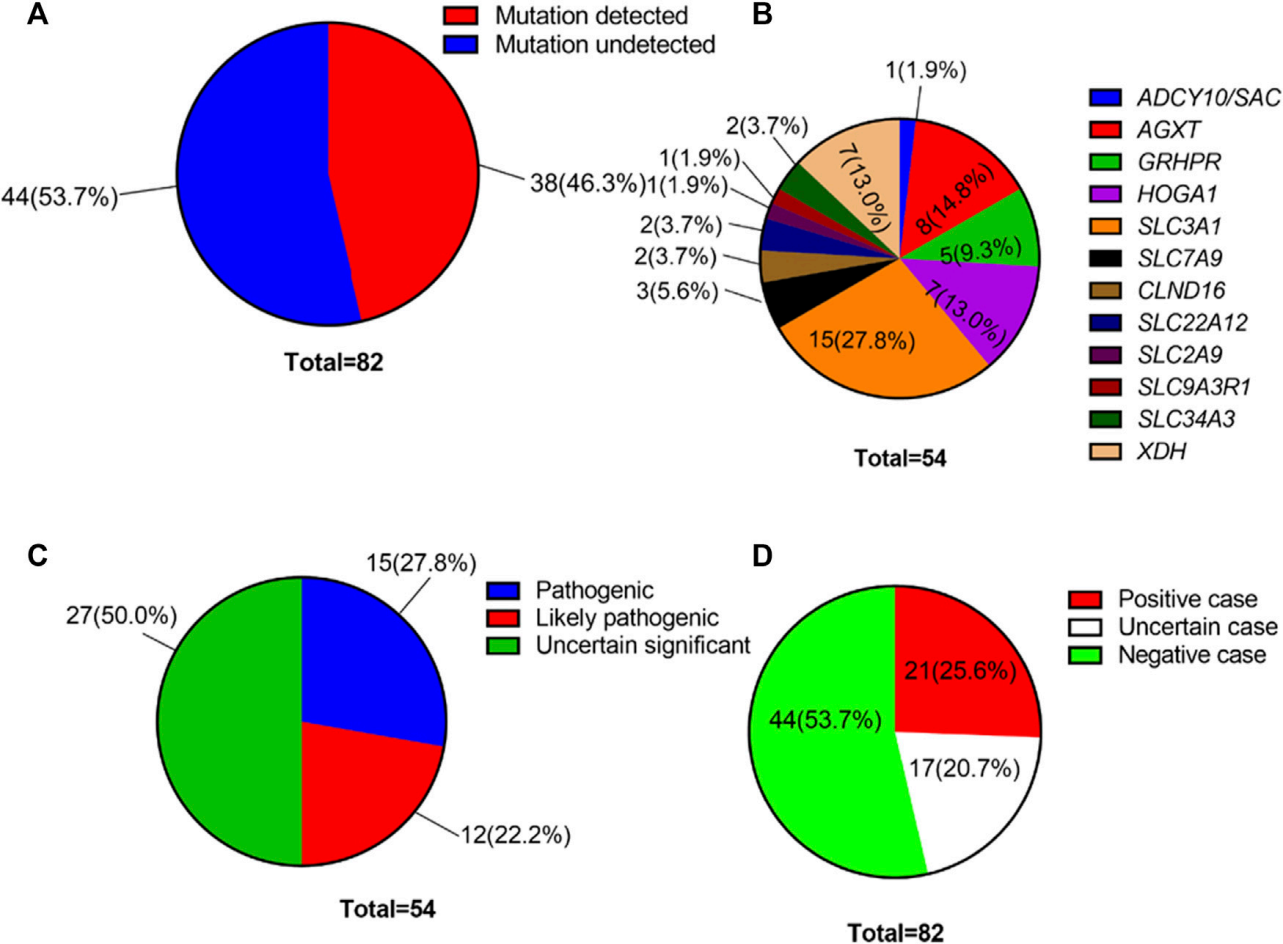
**(A)** Overall genetic mutation detection percentage in our cohort. **(B)** Variant frequency distribution of 12 UL-related genes. **(C)** Distribution of the number of genetic variants by pathogenicity. **(D)** Distribution of the number of affected individuals with UL by variant pathogenicity.

**FIGURE 2:**
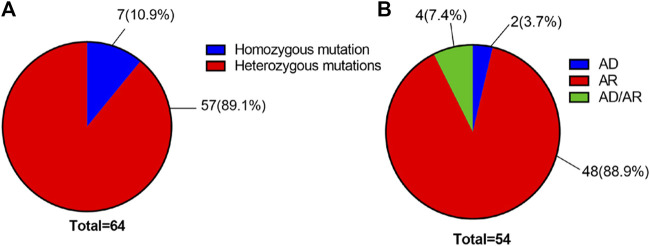
**(A)** Distribution of the number of all variants by genotype. **(B)** Distribution of the number of all variants with UL by the inheritance pattern.

**FIGURE 3:**
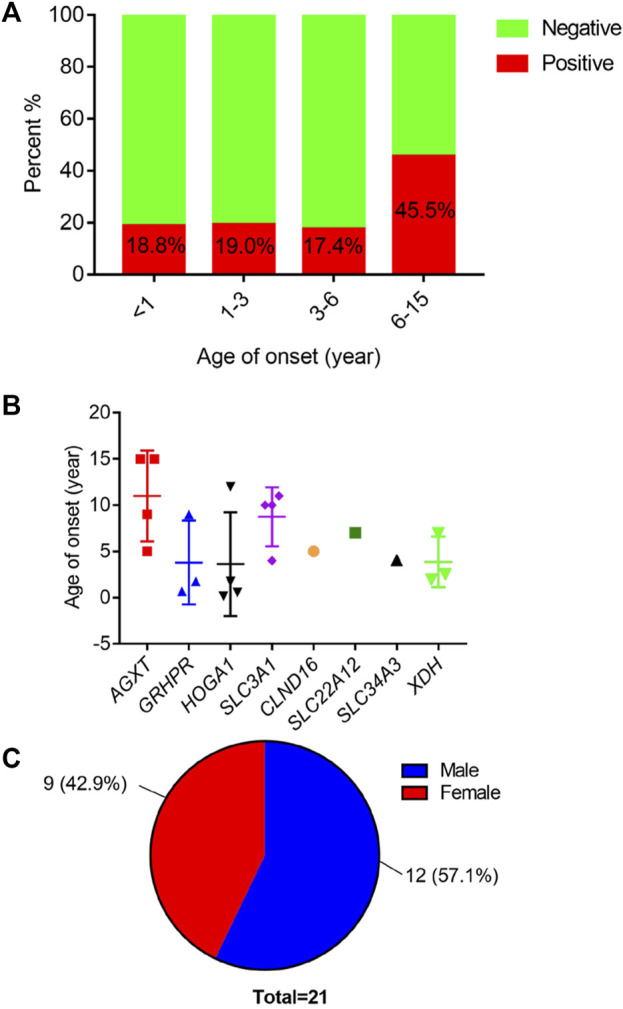
**(A)** Distribution of positive cases by age of onset. **(B)** Distribution of age of onset across mutated causative genes. **(C)** Distribution of the number of positive individuals by gender.

Using high-throughput ES of 30 UL-related genes in 82 patients, we detected genetic variants in 38 of 82 individuals (46.3%) (Figure 1A). A total of 54 mutations were identified in 12 of 30 UL-related genes: one variant (1.9%) in *ADCY10/SAC*, eight variants (14.8%) in *AGXT*, five variants (9.3%) in *GRHPR*, seven variants (13.0%) in *HOGA1*, one variant (1.9%) in *SLC2A9*, two variants (3.7%) in *SLC22A12*, two variants (3.7%) in *SLC34A3*, fifteen variants (27.8%) in *SLC3A1*, two variants (3.7%) in *CLND16*, three variants (5.6%) in *SLC7A9*, one variant (1.9%) in *SLC9A3R1*, and seven variants (13.0%) in *XDH* (Figure 1B; Supplementary Table S1). According to the ACMG International Guidelines Classification Standard, 15 pathogenic mutations were detected in six recessive genes in 13 individuals: *AGXT* (three individuals), *GRHPR* (three individuals), *HOGA1* (three individuals), *CLDN16* (one individual), *SLC22A12* (one individual), and *SLC3A1* (two individuals). Only c.457C>T p.(Gln153*) and c.864_865del p.(Val289fs) in *GRHPR* and c.834G>A (p.Ala278 = ) in *HOGA1*, which were identified as pathogenic mutations, were homozygous, while the others were heterozygous (Table 2; Figure 1C). Twelve mutations classified as likely pathogenic were detected in four genes in 11 individuals: *AGXT* (three individuals), *SLC34A*3 (one individual), *SLC3A1* (four individuals), and *XDH* (three individuals) (Table 3; Figure 1C). Patients 10, 25, and 33 had pathogenic and likely pathogenic mutations simultaneously, so the potential molecular diagnosis of UL was initially made in 21 of 82 individuals (25.6%) (Figure 1D). The same mutations that were detected in two individuals were: *AGXT*, c.823_824dup (p.Ser275Argfs*38); *HOGA1*, c.769T>G p.(Cys257Gly) and c.554C>T(p.Thr185Met), c.715G>A p.(Val239Ile); *XDH*, c.2006G>C (p.Gly669Ala); *GRHPR*, c.864_865del p.(Val289Aspfs); SLC3A1, c.817T>C p.(Cys273Arg) and c.283G>A p.(Ala95Thr); *SLC7A9*, c.878T>C p.(Phe293Ser); and *ADCY10*, c.2996A>G p.(His999Arg). Therefore, the total frequency of detected mutations was 64, of which 57 (89.1%) were heterozygous, and only seven (10.9%) were homozygous (Figure 2A). In addition, uniparental disomy of chromosome 2 was identified in patient P26, who was homozygous for the c.1320G>T (p.Trp440Cys) mutation in *SLC3A1* and c.2006G>C (p.Gly669Ala) in *XDH*. The proband’s father was heterozygous for mutations that were absent in the mother’s DNA. In addition, 48 mutations were inherited in an autosomal recessive (AR) mode of inheritance, two mutations were autosomal dominant (AD), and the combined dual inherited mode of AD/AR was identified in four mutations (Figure 2B). Groups of age <1 year old (50%, 8/16) and 6–15 years old (63.6%, 14/22) exhibited most genetic variants (Supplementary Figure S1A). Variants in dominant genes *ADCY10* and *SLC9A3R1* were mainly identified in probands with an earlier age of onset compared to autosomal recessive/hybrid mode mutations (Supplementary Figure S1B). Male patients accounted for 68.4% of the cases in which genetic mutations were detected, while the proportion of female patients was 31.6% (Supplementary Figure S1C). After exclusion of uncertain variants, the distribution of the age of onset in probands in positive cases is shown in Figure 3A, which demonstrates that genetic diagnoses were made most frequently in the 6–15-year-old group (45.5%, 10/22). The median age of onset across all positive cases was 5 years, ranging from 0.17 to 15 years. The youngest proband had *HOGA1* mutations, and the oldest proband was in the AGXT group. Variants in *HOGA*1 were identified in younger probands (Figure 3B). Male patients accounted for 57.1% of the genetic sequencing-positive cases, while the proportion of female patients was 42.9% (Figure 3C).

**TABLE 2:** Pathogenic mutations of six genes detected in 13 individuals.

Gene symbol	Individual	Status	Mutation	Genetic diagnosis	Mode	Mutation origin	
						Father	Mother
*AGXT*	P10	Heter	c.679_680+2del	PH I	AR	Heter	-
	P25	Heter	c.823_824dup (p.Ser275Argfs*38)	PH I	AR	Heter	-
	P38	Heter	c.33dup(p.Lys12Glnfs*156)	PH I	AR	-	Heter
	P38	Heter	c.823_824dup (p.Ser275Argfs*38)	PH I	AR	Heter	-
*CLDN16*	P36	Heter	c.324+1G>C p.?	FHHNC	AR	Heter	-
	P36	Heter	c.646C>T p.(Arg216Cys)	FHHNC	AR	-	Heter
*GRHPR*	P19	Heter	c.295C>T p.(Arg99*)	PH II	AR	Heter	-
	P28	Homo	c.457C>T p.(Gln153*)	PH II	AR	Heter	Heter
	P32	Homo	c.864_865del p.(Val289fs)	PH II	AR	Heter	Heter
*HOGA1*	P11	Heter	c.834_834+1delinTT	PH III	AR	-	Heter
	P20	Heter	c.769T>G p.(Cys257Gly)	PH III	AR	-	Heter
	P37	Homo	c.834G>A(p.Ala278=)	PH III	AR	Heter	Heter
*SLC22A12*	P29	Heter	c.506+1G>A p.?	Hypouricemia type 1	AR	Heter	-
*SLC3A1*	P33	Heter	c.1113C>A p.(Tyr371*)	Cystinuria	AD/AR	-	Heter
	P1	Heter	c.766-2A>C p.?	Cystinuria	AD/AR	Heter	-
	P1	Heter	c.1011G>A p.(Pro337=)	Cystinuria	AD/AR	-	Heter

Heter, heterozygote; Homo, homozygote; AR, autosomal recessive; AD, autosomal dominant; PH, primary hyperoxaluria; FHHNC, familial hypomagnesemia with hypercalciuria and nephrocalcinosis.

**Table 3:** Likely pathogenic mutations of four genes detected in 11 individuals.

Gene symbol	Individual	Status	Mutation	Genetic diagnosis	Mode	Mutation origin	
						Father	Mother
*AGXT*	P8	Heter	c.568G>A (p.Gly190Arg)	PH I	AR	Heter	-
	P10	Heter	c.506_510del insGCAGGT	PH I	AR	-	Heter
	P25	Heter	c.32C>G (p.Pro11Arg)	PH I	AR	-	Heter
*SLC34A3*	P5	Heter	c.410C>T p.(Thr137Met)	HHRH	AR	Heter	-
*SLC3A1*	P31	Heter	c.436C>T p.(Gln146*)	Cystinuria	AD/AR	Heter	-
	P33	Heter	c.183delC p.(Val62Serfs)	Cystinuria	AD/AR	Heter	-
	P7	Heter	c.1772_1773del p.(Arg591fs)	Cystinuria	AD/AR	-	Heter
	P26^a^	Homo	c.1320G>T(p.Trp440Cys)	Cystinuria	AD/AR	Heter	-
*XDH*	P17	Heter	c.1253_1256del	Xanthinuria type I	AR	Heter	-
	P21	Heter	c.472C>T (p.Gln158*)	Xanthinuria type I	AR	-	Heter
	P21	Heter	chr2:31621429-31624204 del	Xanthinuria type I	AR	Heter	-
	P23	Heter	c.2198-1G>C		AR	Heter	-

Heter, heterozygote; Homo, homozygote; AR, autosomal recessive; AD, autosomal dominant; PH, primary hyperoxaluria; HHRH, hereditary hypophosphatemic rickets with hypercalciuria. ^a^A uniparental disomy (UPD) of chromosome 2 was identified in this proband; the proband’s father was heterozygous for these mutations.

As shown in Table 4, the results of ES indicated that 6 novel genetic mutations not reported in previous studies were detected in 6 patients with UL. A heterozygous mutation (c.929C>A) in exon 7 of *SLC2A9* resulted in an amino acid change from alanine to aspartic acid. Three missense mutations in SLC3A1 yielded cystine stone formation: c.1216G>T p.(Asp406Tyr), c.1772_1773del p.(Arg591fs), and c.1320G>T (p.Trp440Cys). The *XDH*-causing genetic mutation c.2006G>C (p.Gly669Ala) in *XDH* has not been reported but has been demonstrated to appear in a heterozygous state in one patient and in a homozygous state in another patient with a cystine stone. Moreover, a heterozygous truncated mutant (chr2:31621429-31624204 del) was detected in *XDH* in the heterozygous state.

**TABLE 4:** Six novel variants identified by exome sequencing.

Gene symbol	Individual	Status	Mutation	Genetic diagnosis	Stone composition	Mode	Mutation origin	
							Father	Mother
*SLC2A9*	P34	Heter	c.929C>A p.(Ala310Asp)	Hypouricemia type 2	Carbonate apatite	AD/AR	Heter	-
*SLC3A1*	P24	Heter	c.1216G>T p.(Asp406Tyr)	Cystinuria	Cystine	AR	Heter	-
	P7	Heter	c.1772_1773del p.(Arg591fs)	Cystinuria	Cystine	AR	-	Heter
	P26^a^	Homo	c.1320G>T(p.Trp440Cys)	Cystinuria	Cystine	AD/AR	Heter	-
*XDH*	P26^a^	Homo	c.2006G>C (p.Gly669Ala)	Xanthinuria type I	Cystine	AR	Heter	-
	P29	Heter	c.2006G>C p.(Gly669Ala)	Xanthinuria type I	Cystine	AR	-	Heter
	P21	Heter	chr2:31621429-31624204 del	Xanthinuria type I	Xanthine	AR	Heter	-

Heter, heterozygote; Homo, homozygote; AR, autosomal recessive; AD, autosomal dominant, PH, primary hyperoxaluria.

^a^ A uniparental disomy (UPD) of chromosome 2 was identified in this proband; the proband’s father was heterozygous for these mutations.

In this study, multiple online bioinformatic analysis tools were employed to perform pathogenicity and function prediction of the novel mutations, as demonstrated in Table 5: MutationTaster and Franklin by Genoox were used to obtain general information about the variants; PolyPhen-2, SIFT, and PROVEAN are mature predictive software programs for potential protein-level function. Except for *SLC2A9* c.929C>A p.(Ala310Asp) classified as “polymorphism,” other variants were predicted to be “disease causing” by MutationTaster. Furthermore, *SLC3A1* variant c.1216G>T p.(Asp406Tyr) was predicted to exert pathogenic impact by Mutation Taster, PolyPhen-2, SIFT, PROVEAN and Franklin by Genoox tools, while it was classified under “uncertain” group according to ACMG classification standard. *XDH* variant c.2006G>C (p.Gly669Ala) was described as diseasing-causing by Mutation Taster, PolyPhen-2, PROVEAN but was classified as uncertain by Franklin by Genoox and ACMG guideline.

**TABLE 5:** Pathogenicity of six novel mutations in 30 stone-causing genes.

Gene symbol	Mutation	MutationTaster (Probability)	PP2 HumVar (score)	PROVEAN (score)	SIFT (score)	Genoox (score)	ACMG classification
*SLC2A9*	c.929C>A p.(Ala310Asp)	PM (0.9999)	BN (0.004)	Neu (1.60)	Tol (0.681)	BN (0.14)	VUS (PM2,BP4)
*SLC3A1*	c.1216G>T p.(Asp406Tyr)	DC (0.9999)	PD (0.991)	Del (−4.77)	Dam (0.006)	Del (0.87)	VUS (PM2,PP3,PP2)
	c.1772_1773del p.(Arg591fs)	DC (1.0000)	NA	NA	NA	NA	LP (PVS1,PM2)
	c.1320G>T(p.Trp440Cys)	DC (0.9999)	PD (1.000)	Del (−8.60)	Dam (0.002)	Del (0.99)	LP (PP3,PM2,PP2)
*XDH*	c.2006G>C (p.Gly669Ala)	DC (0.9999)	PD (0.980)	Del (−4.97)	Tol (0.897)	UC (0.47)	VUS (PM2)
	chr2:31621429-31624204 del	DC (1.0000)	NA	NA	NA	NA	LP (1A,2B,3A,4O)

DC, disease causing; PM, polymorphism; PD, probably damaging; BN, benign; Del, deleterious; Tol, tolerated; Neu, neutral; Dam, damaging; UC, uncertain; NA, not applicable for deletion or frameshift mutation; VUS, variant of uncertain significance; P, pathogenic; LP, likely pathogenic.

### 3.3 Conformity between genetic variants and clinical metabolic analysis

Conformity analysis between the mutational findings and metabolic evaluation was conducted in 14 mutation-detection-positive individuals with complete stone component analysis and positive ES results. Hyperoxaluria-related mutations were detected in nine patients (three *AGXT* defects, three *GRHPR* defects, and three *HOGA1* defects), and calcium oxalate stones were found in eight of these patients as a result of stone sample evaluation. Four of the five patients (80%) with cystinuria-related gene *SLC3A1* mutations were confirmed to have cystine stones (Table 6).

**TABLE 6:** Conformity between UL-causing genetic mutations and stone composition.

Molecular diagnosis	Total,n	Stone composition	Conform *n* (%)
Overall	14		
*AGXT* defect	3	Calcium oxalate (3)	3 (100.0%)
*GRHPR* defect	3	Calcium oxalate (3)	3(100.0%)
*HOGA1* defect	3	Calcium oxalate (2), Carbonate apatite (1)	2 (66.7%)
*SLC3A1* defect	5	Cystine (4), Calcium oxalate (1)	4 (80.0%)

## 4 Discussion

Pediatric UL often frustrates pediatricians and urologists because of its early onset, high recurrence rate, relatively poor prognosis, and treatment ambiguity. In the clinical setting, this condition is often overlooked or misdiagnosed. Recently, several genetic causes of UL have been identified. At least 30 genes have been shown to cause the monogenic forms of UL *via* autosomal-dominant, autosomal-recessive, or X-linked transmission (Halbritter et al., 2015). Previously, monogenic ULs were considered to have an incidence rate of less than 2%. However, growing evidence suggests that they might be more common than expected (Singh et al., 2022). Current molecular genetic diagnostic techniques make screening for hereditary diseases feasible. Next-generation sequencing combined with a designed multigene panel can be conducted for a comprehensive analysis of specific diseases or disease groups with a faster and more precise process (Ziyadov et al., 2021).

In this study, ES was performed on a cohort of 82 pediatric patients with UL. We sequenced the exon regions of 30 genes known to cause the monogenic form of UL and identified a causative mutation in 38 of 82 individuals (46.3%). This rate of identifying causative mutations in children with UL is higher than that reported by Braun et al., who found a positive mutation rate of 16.8% in a pediatric NL/nephrocalcinosis (NC) cohort (Braun et al., 2016). This was also higher than that in a previous report that found a monogenic cause in 29.4% of the families presenting with NL or NC at the age of onset less than 25 years (Daga et al., 2018). Halbritter et al. (2015) also observed a relatively low percentage of monogenic cases in the pediatric NL/NC subgroup (20.8%). In our study, the causative mutation detection rate plunged to 25.6% after excluding patients with uncertain variants, which is slightly higher than previous reports. All these individuals were recruited from a referral center for the management of pediatric UL in China, where early onset, severe, and stone-recurrent cases present more frequently than in other national or local hospitals. Moreover, only UL cases were enrolled in our research rather than including NL/NC together, and UL cases usually sustain higher genetic abnormality rates and more severe clinical symptoms than NC. Therefore, selective bias in our cohort and different inclusion criteria may have contributed to the differences in the genetic landscapes among the multiple cohort studies.

The most commonly mutated gene in our cohort was *SLC3A*1, which encodes a 685-amino acid glycoprotein rBAT that plays a role in the reabsorption of cystine, ornithine, lysine, and arginine as subunits of heterodimeric amino acid transporters and proximal tubular transporters (Yan et al., 2020). Mutations in *SLC3A*1 lead to high urinary cystine excretion, precipitation, and crystal formation, resulting in an increased risk of cystine stone formation (Hoppe and Martin-Higueras, 2020). Heterozygous carriers of the *SLC3A1* mutation normally show an increased urinary cystine excretion pattern (Eggermann et al., 2012). We detected 15 genetic mutations in *SLC3A1*, including three pathogenic mutations and three novel variants. Only the novel mutation, c.1320G>T (p.Trp440Cys), was detected in the homozygous state. This mutation has never been reported in the literature, whereas other mutations were found in heterozygote carriers. Some undetected alleles or loci may underlie the heterozygous carriers of *SLC3A1* mutations that cause urolithiasis. The Met467Thr mutation in *SLC3A1* is the most frequent in European and North American populations (Shen et al., 2017). However, this mutation was not found in our results, indicating genetic variant heterogeneity among ethnicities.

Age distribution analysis of monogenetic mutations in the UL-causing gene panel was performed in our cohort. Individuals with autosomal recessive variants typically manifest earlier onset in life than dominant mutation carriers, such as those with hereditary polycystic kidney disease and previous inherited UL reports (Braun et al., 2016; Ghata and Cowley, 2017). Interestingly, we found that cases with the age of onset ranging from 6 to 15 years showed a high positive causative mutation detection rate, but the overall sample size was limited, and a larger number of cases needs to be included in further studies. The prevalence and heritability of urolithiasis vary according to sex. Traditionally, male cases predominated the sex distribution, with a ratio of 3:1 to female cases (Cicerello et al., 2021). Two twin studies reported that the heritability of kidney stones was at an estimated rate of 56%–57% in male twins, while the rate for female twins was only 46% (Halbritter, 2021). Notably, in pediatric UL, a higher prevalence was observed among boys in the first decade of life and among girls in the second decade of life, and girls represented 70% of the pediatric UL population in general (Van Batavia and Tasian, 2016). Fang et al. (2021) reported 39.9% of idiopathic stone patients were male in his hospitalized pediatric UL cohort. Our findings demonstrated a similar sex proportion to previous studies, with a ratio of 2.03/1 (male/female) in all subjects and 1.3/1 in positive individuals, suggesting that male patientss still accounted for most of the pediatric stone probands and needed more urgent early intervention. However, only six patients were older than 10 years in this study, and the inheritability of UL in terms of sex distribution during the second decade of life remains undetermined.

A pivotal step in ES analysis is the identification of potentially pathogenic mutations and benign variants. All mutations identified in our study were evaluated for pathogenicity and allele frequency by cross-checking them against public data banks, such as HGMD and ClinVar, and predicted using the ACMG criteria. The pathogenicity of 27 (50.0%) of the 54 detected variants was uncertain based on the ACMG classification, whereas 6 variants were novel and had not been previously identified. Two novel variants in *SLC3A1*, c.1320G>T(p.Trp440Cys), and c.1772_1773del p.(Arg591fs), were predicted to be likely pathogenic by ACMG classification. MutationTaster (http://www.mutationtaster.org/) yielded consistent results, implying that a frameshift mutant resulting in truncated proteins was likely to cause disease. c.2006G>C (p.Gly669Ala) (two individuals) in *XDH* was the most common novel mutation identified in our study. Although they were identified in an ACMG “uncertain” subgroup, multiple *in silico* analysis tools predicted it as potentially disease-causing. Notably, this variant and c.878T>C p.(Phe293Ser) in SLC7A9 were detected in a compound heterozygous state in patient P29, a cystic stone case who experienced recurrent nephrolithiasis three times. Therefore, these novel mutations broaden our knowledge of known pathogenic gene mutations implicated in UL. Novel mutational pathogenicity that is not yet recognized or has obscured clinical significance will be identified through genetic profiling mediated by high-throughput sequencing and complementary functional studies.

Normally, a genetic diagnosis must be consistent with the patient’s actual metabolic pattern abnormalities to confirm inherited UL. Thus, a comparative analysis between the metabolic evaluation and genetic results in positive cases was performed to confirm the consistency of our study. The hyperoxaluria-related variant was detected in eight of the nine (88.9%) patients in whom calcium oxalate was confirmed by stone component analysis. The *AGXT* and *GRHPR* defects showed 100% (3/3) conformity with the stone analysis results, whereas the *HOGA1* defect sustained a conformity rate of 66.7%. In the cystine stone subgroup, a high level of conformity was also observed in the *SLC3A1* (80.0%) variants. Our finding demonstrated ES yielded high diagnostic precision for genetic UL with metabolic abnormalities.

This study has some limitations. The relatively small number of patients, single ethnic background, and nature of the single-center study limit the universality and comprehensiveness of the interpretation and application of the results. In some studies, a female predominance was found in the pediatric UL population, especially in the second decade of life. The gender ratio in our study presents a male predominance. Therefore, a large, multicenter population-based study may be needed to determine this demographic factor in the Chinese Han population. The technical limitations of ES, which include uncovering untranslated regions and undetected complex deletion-insertion variants, compromise the reliability and credibility of high-throughput sequencing. Novel causative mutations may be missed because the gene set we selected to study may not cover some genes that cause urolithiasis/nephrocalcinosis phenocopies rather than urinary stones. Although the current results may be compromised by such selection bias, the actual prevalence of monogenic UL in children will be refined through further research. Additionally, obtaining sufficient stone samples for component analysis is difficult due to specific surgical interventions, such as extracorporeal shockwave lithotripsy or undersized calculus that result in incomplete metabolic evaluation. Finally, further functional studies are required to systematically verify the candidate pathogenic genes.

## 5 Conclusion

In this study, we analyzed the genotypes and phenotypes of pediatric urolithiasis in 82 patients and identified stone-causing genetic defects in 8 genes in 25.6% of the individuals. Six novel genetic mutations were identified, and a high degree of association between genotype and phenotype was observed. Chinese Han UL pediatric patients exhibited high genetic mutation frequency and early onset. ES is a cost-effective and reliable tool for the molecular diagnosis of monogenetic urolithiasis. Genetic screening should be applied in pediatric UL patients because individual genetic profiles may facilitate the personalization of treatment plans, monitoring treatment responses, and target drug development. This study identified the monogenic cause of pediatric UL, improved our understanding of the underlying molecular mechanisms, and provided novel genetic targets for further research.

## Data Availability

The data presented in the study are deposited in the National Genomics Data Center, accession number “PRJCA015789”.
