# Supplementary material for: Use of whole-exome sequencing to identify novel monogenic gene mutations and genotype–phenotype correlations in Chinese Han children with urolithiasis
Source: Front Genet. 2023 Apr 18;14:1128884. doi: 10.3389/fgene.2023.1128884 (PMC10152365; doi:10.3389/fgene.2023.1128884)
Supplement: Supplementary file 1 [file Table1.DOCX]

Supplementary Material

Supplemental Table 1. Molecular diagnosis by WES in 12 UL disease-causing genes from 38 pediatric cases

| Individual | Gender | Age (year) | Gene Symbol | Status | Mutation | Genetic diagnosis | Mode | Mutation origin | |
| --- | --- | --- | --- | --- | --- | --- | --- | --- | --- |
|  |  |  |  |  |  |  |  | Father | Mother |
| P1 | Male | 11 | SLC3A1 | Heter | c.766-2A>C p.? | Cystinuria | AR | Heter | - |
|  |  |  | SLC3A1 | Heter | c.1011G>A p.(Pro337=) | Cystinuria | AR | - | Heter |
| P2 | Male | 11 | XDH | Heter | c.1042G>A p.(Val348Ile) | Xanthinuria type I | AR | - | Heter |
| P3 | Male | 3.58 | ADCY10 | Heter | c.2996A>G p.(His999Arg) | Hypercalciuria | AD | - | Heter |
|  |  |  | GRHPR | Heter | c.16C>T p.(Leu6Phe) | PH II | AR | Heter | - |
| P4 | Male | 0.5 | SLC3A1 | Heter | c.242G>T p.(Arg81Leu) | Cystinuria | AR | Heter | - |
| P5 | Male | 4 | SLC34A3 | Heter | c.410C>T p.(Thr137Met) | HHRH | AR | Heter | - |
| P6 | Male | 0.42 | XDH | Heter | c.2414G>A p.(Arg805Gln) | Xanthinuria type I | AR | Heter | - |
| P7 | Female | 10 | SLC3A1 | Heter | c.1337G>A p.(Gly446Asp) | Cystinuria | AR | Heter | - |
|  |  |  | SLC3A1 | Heter | c.1772_1773del p.(Arg591fs) | Cystinuria | AR | - | Heter |
| P8 | Female | 15 | AGXT | Heter | c.568G>A (p.Gly190Arg) | PH I | AR | Heter | - |
|  |  |  | AGXT | Heter | c.1147_1159 del(p.Leu384Profs*141) | PH I | AR | - | Heter |
| P9 | Male | 1.58 | SLC7A9 | Heter | c.829G>A p.(Val277Met) | Cystinuria | AR/AD | - | Heter |
| P10 | Male | 15 | AGXT | Heter | c.679_680+2del | PH I | AR | Heter | - |
|  |  |  | AGXT | Heter | c.506_510del insGCAGGT | PH I | AR | - | Heter |
| P11 | Female | 12 | HOGA1 | Heter | c.834_834+1delinTT | PH III | AR | - | Heter |
|  |  |  | HOGA1 | Heter | c.811C>T (p.Arg271Cys) | PH III | AR | Heter | - |
| P12 | Male | 1.25 | SLC7A9 | Homo | c.878T>C p.(Phe293Ser) | Cystinuria | AR/AD | Heter | Heter |
| P13 | Female | 0.58 | HOGA1 | Heter | c.554C>T(p.Thr185Met) | PH III | AR | Heter | - |
|  |  |  | HOGA1 | Heter | c.793G>A(p.Ala265Thr) | PH III | AR | - | Heter |
| P14 | Male | 2 | SLC3A1 | Heter | c.817T>C p.(Cys273Arg) | Cystinuria | AR | Heter | - |
|  |  |  | SLC3A1 | Heter | c.1355G>T p.(Arg452Leu) | Cystinuria | AR | Heter | - |
| P15 | Male | 3 | SLC22A12 | Heter | c.679G>A p.(Ala227Thr) | RHUC1 | AR | Heter | - |
| P16 | Male | 12 | SLC3A1 | Heter | c.197C>T p.(Ala66Val) | Cystinuria | AR | - | Heter |
| P17 | Male | 7 | XDH | Heter | c.1253_1256del | Xanthinuria type I | AR | Heter | - |
| P18 | Male | 0.75 | HOGA1 | Heter | c.715G>A p.(Val239Ile) | PH III | AR | - | Heter |
|  |  |  | SLC34A3 | Heter | c.545G>A p.(Arg182Gln) | HHRH | AR | - | Heter |
| P19 | Male | 1.75 | GRHPR | Heter | c.295C>T p.(Arg99*) | PH II | AR | Heter | - |
|  |  |  | GRHPR | Heter | c.864_865del p.(Val289Aspfs) | PH II | AR | - | Heter |
| P20 | Male | 0.17 | HOGA1 | Heter | c.769T>G p.(Cys257Gly) | PH III | AR | - | Heter |
| P21 | Female | 2.58 | XDH | Heter | c.472C>T (p.Gln158*) | Xanthinuria type I | AR | - | Heter |
|  |  |  | XDH | Heter | chr2:31621429-31624204 del | Xanthinuria type I | AR | Heter | - |
| P22 | Male | 6.25 | HOGA1 | Heter | c.554C>T p.(Thr185Met) | PH III | AR | - | Heter |
| P23 | Male | 2 | XDH | Heter | c.2198-1G>C | Xanthinuria type I | AR | Heter | - |
| P24 | Male | 10 | SLC3A1 | Heter | c.470T>C p.(Ile157Thr) | Cystinuria | AR | - | Heter |
|  |  |  | SLC3A1 | Heter | c.1216G>T p.(Asp406Tyr) | Cystinuria | AR | Heter | - |
|  |  |  | SLC7A9 | Heter | c.338_340del p.（ S113del） | Cystinuria | AR/AD | - | Heter |
| P25 | Female | 5 | AGXT | Heter | c.823_824dup (p.Ser275Argfs*38) | PH I | AR | Heter | - |
|  |  |  | AGXT | Heter | c.32C>G (p.Pro11Arg) | PH I | AR | - | Heter |
| P26^a^ | Male | 0.5 | SLC3A1 | Homo | c.1320G>T(p.Trp440Cys) | Cystinuria | AR | Heter | - |
|  |  |  | XDH | Homo | c.2006G>C (p.Gly669Ala) | Xanthinuria type I | AR | Heter | - |
| P27 | Male | 0.58 | HOGA1 | Homo | c.769T>G(p.Cys257Gly) | PH III | AR | Heter | Heter |
| P28 | Female | 0.67 | GRHPR | Homo | c.457C>T p.(Gln153*) | PH II | AR | Heter | Heter |
| P29 | Male | 7 | SLC7A9 | Heter | c.878T>C p.(Phe293Ser) | Cystinuria | AR/AD | - | Heter |
|  |  |  | SLC22A12 | Heter | c.506+1G>A p.? | RHUC1 | AR | Heter | - |
|  |  |  | XDH | Heter | c.2006G>C p.(Gly669Ala) | Xanthinuria type I | AR | - | Heter |
|  |  |  | AGXT | Heter | c.589C>T p.(Arg197Trp) | PH I | AR | Heter | - |
| P30 | Female | 1 | ADCY10 | Heter | c.2996A>G p.(His999Arg) | Hypercalciuria | AD | - | Heter |
|  |  |  | HOGA1 | Heter | Exon6 c.715G>A p.(Val239Ile) | PH III | AR | Heter | - |
| P31 | Male | 4 | SLC3A1 | Heter | c.817T>C p.(Cys273Arg) | Cystinuria | AR | - | Heter |
|  |  |  | SLC3A1 | Heter | c.436C>T p.(Gln146*) | Cystinuria | AR | Heter | - |
|  |  |  | SLC3A1 | Heter | c.283G>A p.(Ala95Thr) | Cystinuria | AR | Heter | - |
|  |  |  | GRHPR | Heter | c.512G>A p.(Arg171His) | PH II | AR | Heter | - |
| P32 | Female | 9 | GRHPR | Homo | c.864_865del p.(Val289fs) | PH II | AR | Heter | Heter |
| P33 | Male | 10 | SLC3A1 | Heter | c.1113C>A p.(Tyr371*) | Cystinuria | AR | - | Heter |
|  |  |  | SLC3A1 | Heter | c.183delC p.(Val62Serfs) | Cystinuria | AR | Heter | - |
| P34 | Female | 3.42 | SLC2A9 | Heter | c.929C>A p.(Ala310Asp) | RHUC2 | AR/AD | Heter | - |
| P35 | Male | 1 | SLC9A3R1 | Heter | c.539G>A p.(Arg180Gln) | NPHLOP2 | AD | - | Heter |
| P36 | Male | 5 | CLDN16 | Heter | c.324+1G>C p.？ | FHHNC | AR | Heter | - |
|  |  |  | CLDN16 | Heter | c.646C>T p.(Arg216Cys) | FHHNC | AR | - | Heter |
|  |  |  | SLC3A1 | Heter | c.283G>A p.(Ala95Thr) | Cystinuria | AR | Heter | - |
| P37 | Female | 1.75 | HOGA1 | Homo | c.834G>A(p.Ala278=) | PH III | AR | Heter | - |
| P38 | Female | 9 | AGXT | Heter | c.33dup(p.Lys12Glnfs*156) | PH I | AR | - | Heter |
|  |  |  | AGXT | Heter | c.823_824dup (p.Ser275Argfs*38) | PH I | AR | Heter | - |

Heter: heterozygote; Homo: homozygote; AR: Autosomal recessive; AD: Autosomal dominant, PH, Primary hyperoxaluria; HHRH, Hereditary hypophosphatemic rickets with hypercalciuria; RHUC, Renal hypouricemia; NPHLOP2, Hypophosphatemic nephrolithiasis/osteoporosis-2; FHHNC, Familial hypomagnesemia with hypercalciuria & nephrocalcinosis; ^a^ a uniparental disomy (UPD) of chromosome 2 was identified in this proband, the proband's father was heterozygous for these mutations.

**
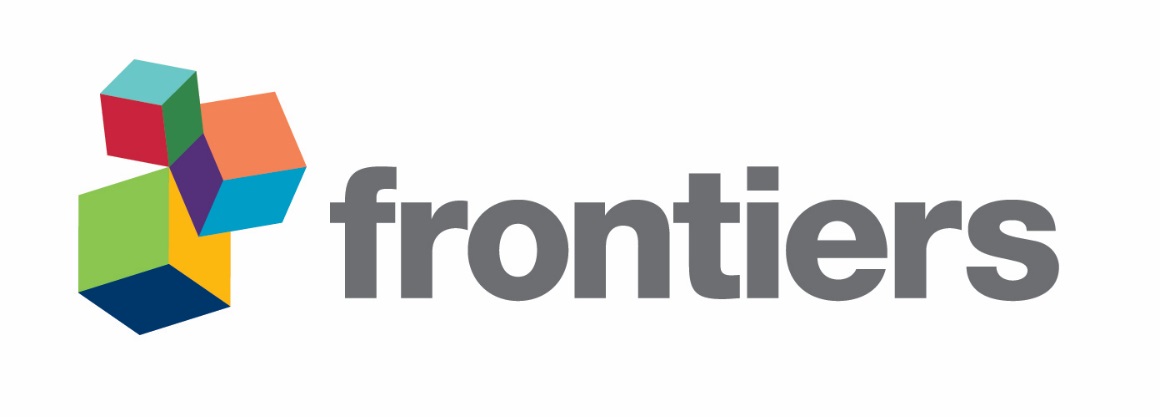
**
